# Association between serum 25-hydroxyvitamin D3 level and cognitive impairment in older chronic kidney disease patients

**DOI:** 10.1038/s41598-024-63350-y

**Published:** 2024-05-30

**Authors:** Jialing Zhang, Aihua Zhang

**Affiliations:** 1 Department of Nephrology, Xuanwu Hospital, Capital Medical University, 45 Changchun Street, Xicheng District, Beijing, China; 2 The National Clinical Research Center for Geriatric Disease, Xuanwu Hospital, Capital Medical University, Beijing, China

**Keywords:** Kidney diseases, Neurological disorders, Nutrition disorders

## Abstract

This study aims to examine whether hypovitaminosis D was associated with cognitive impairment among chronic kidney patients with different level of albuminuria. This population-based cross-sectional study was conducted on elderly (over 60 years old) with urine albumin to creatinine ratio (UACR) ≥ 30 mg/g from 2011 to 2014 in the US National Health and Nutrition Examination Survey (NHANES). Cognitive function was assessed by the Consortium to Establish a Registry for Alzheimer’s Disease Word List Learning (CERAD). Subjects were divided into 2 groups according to the absence or presence of cognitive impairment and a propensity score matching (PSM) was further conducted. The association was assessed with Spearman correlation and logistic regression analysis. The positive association of 25-hydroxyvitamin D3 (25(OH)D3) and cognitive score was presented. PSM analysis revealed that a higher level of 25(OH)D3 correlated to a better cognitive function in CKD patients with albuminuria, especially in patients with 30 mg/g ≤ UACR < 300 mg/g. This study indicated that a low 25(OH)D3 level was associated with poor cognitive performance, especially in patients with microalbuminuria. Thus, early diagnosis of vitamin D insufficiency and an effective intervention might be a useful therapeutic strategy to prevent cognitive decline in patients with the progression of renal dysfunction.

## Introduction

Chronic kidney disease (CKD) is a major challenge to public health, associated with an increasing mortality and morbidity^1^. Cognitive impairment is a common complication in individuals with CKD. Both albuminuria and low estimated glomerular filtration rate (eGFR) are associated with elevated risks for cognitive impairment^2,3^. Albuminuria is considered as a measure of kidney damage, quantitated by urine albumin to creatinine ratio (UACR)^4^. Albuminuria (UACR ≥ 30 mg/g) was associated with both worse baseline cognitive function and cognitive decline^5,6^. Participants having an elevated proteinuria probably showed a neurocognitive dysfunction^3,7,8^. Multiple risk factors for cognitive dysfunction in CKD patients were demonstrated, including older age, proteinuria, educational level, hypertension, acidosis, anemia, and uremic milieu^9,10^.

Vitamin D is a kind of fat-soluble seco-steroid for calcium uptake and bone metabolism^11^. Hypovitaminosis D is highly frequent in older adults^12^. The functions of vitamin D to regulate mineral homeostasis, adaptive and innate immunity, and cardiovascular disease in patients with CKD have been proposed^13^. The influence of hypovitaminosis D and cognitive dysfunction was also explored^14,15^. However, other studies showed nonsignificant associations between lower 25-hydroxyvitamin D (25(OH)D) levels and poor cognitive function in patients with Parkinson’s disease^16,17^. The effect of 25(OH)D on domain-specific cognitive performance was also unclear in patients with peritoneal dialysis and hemodialysis^18,19^.

25(OH)D3 is widely applied to assess vitamin D status as a main circulating form of vitamin D. Little is known about the relationship between 25(OH)D3 and the cognitive function in CKD patients. In the present study, we aimed to examine the potential association of serum 25(OH)D3 and the cognitive performance of CKD patients with different level of albuminuria.

## Results

### Participant characteristics

Table 1 showed the characteristics of the patients before propensity score matching. Among 583 older adults, the median age was 71 years, and 49.4% were male. In comparison to the normal cognition function, patients with cognitive impairment were older and had a lower level of calcium, phosphate, and 25(OH)D3. Characteristics of participants were also summarized in the Supplementary Table 1 stratified by quartile of serum 25(OH)D3. Of these patients, age, education level, serum albumin, BUN, calcium, alkaline phosphatase levels were significantly different among four groups according to the quartile of serum 25(OH)D3. The prevalence of hypertension and diabetes significantly differed among different level of 25(OH)D3. There were significant differences in CERAD scores among the four groups.

**Table 1:** Comparison of patient characteristics between patients with and without cognitive impairment before propensity score matching.

	Total (n = 583)	No cognitive impairment (n = 420)	Cognitive impairment (n = 163)	*P*
Age (years)	71 (65–80)	70 (64–78)	74.5 (65.25–80)	0.001
Male (%)	49.4	50.5	56.7	0.175
Race (%)				0.32
Mexican American (%)	10.5	10.2	11.6	
Other Hispanic (%)	9.9	9.5	9.8	
Non-Hispanic White (%)	43.8	45.2	42.1	
Non-Hispanic Black (%)	26.1	27.1	23.2	
Other (%)	41.8	7.6	13.4	
Education (%)				< 0.001
Less than 9th grade (%)	15.2	11.9	26.8	
9 − 11th grade (%)	16	16.7	17.7	
High school graduate (%)	23.2	23.6	26.8	
College or AA degree (%)	23.8	29.4	14.6	
College graduate or above (%)	15.7	18.1	12.8	
CKD stage (%)				0.141
CKD stage 1	25.1	22.1	17.7	
CKD stage 2	39.5	41.4	42.7	
CKD stage 3	27.9	28.8	31.1	
CKD stage 4	5.2	4.5	7.9	
CKD stage 5	2.3	3.1	0.6	
Smoke (%)	33.4	37	35.9	0.803
Drink (%)	57.5	66.3	55.6	0.021
Hypertension (%)	75.4	76	73.8	0.584
Diabetes (%)	41.8	39.8	46.3	0.147
Height (m)	1.64 (1.57–1.72)	1.64 (1.57–1.72)	1.64 (1.56–1.71)	0.354
Weight (kg)	76.3 (66.3–91.5)	77.6 (67.35–93.68)	74.2 (63.85–89.85)	0.067
BMI (kg/m2)	28.12 (24.25–32.66)	28.43 (24.71–33.19)	27.54 (23.85–31.97)	0.085
Hemoglobin (g/dl)	13.5 (12.5–14.5)	13.5 (12.5–14.5)	13.5 (12.3–14.5)	0.783
Albumin (g/l)	42 (39–43)	42 (40–43)	41 (39–43)	0.442
BUN (mg/dl)	11 (6.07–17)	11 (6.07–17)	10 (6.07–16)	0.504
Creatinine (μmol/l)	90.17 (71.6–117.79)	89.28 (71.6–116.69)	91.94 (73.37–119.34)	0.306
Calcium (mmol/l)	2.35 (2.3–2.43)	2.38 (2.3–2.43)	2.33 (2.3–2.43)	0.029
Phosphate (mmol/l)	1.2 (1.1–1.32)	1.2 (1.1–1.32)	1.16 (1.07–1.29)	0.041
Cholesterol (mmol/l)	4.66 (3.91–5.61)	4.66 (3.9–5.62)	4.66 (3.91–5.52)	0.558
Triglyceride (mmol/l)	1.54 (1.04–2.43)	1.5 (1.03–2.38)	1.6 (1.05–2.6)	0.625
Uric acid (mg/dl)	5.9 (4.9–7.2)	5.9 (4.9–7.2)	5.8 (4.7–7.15)	0.378
eGFR (ml/min/1.73m2)	67.05 (51.63–85.1)	67.51 (51.93–85.36)	66.56 (47.86–84.09)	0.469
UACR (mg/g)	69.68 (43.68–170.32)	66.55 (43.84–148.3)	82.86 (42.82–225.65)	0.248
CERAD score	18 (14–21)	20 (17–22)	11 (7–13)	< 0.001
25(OH)D3 (nmol/l)	61.56 (41.84–84.98)	65.16 (43.65–88.68)	54.18 (39.76–72.98)	< 0.001

Data are presented as medians (interquartile ranges) for continuous variables.

*CERAD* Consortium to Establish a Registry for Alzheimer’s Disease Word Learning test, *CKD* Chronic kidney disease, *BMI* Body mass index, *eGFR* Estimated glomerular filtration rate, *UACR* Urea albumin-creatinine ratio, *BUN* Blood urea nitrogen.

### Correlation analysis between cognitive function and various parameters

The Spearman correlation test was performed to explore the potential associated factors for cognition function. As shown in Table 2, we found that age was negatively associated with CERAD score, while BMI, BUN, calcium, phosphate and 25(OH) D3 concentrations were positively associated with good cognitive ability in patients with CKD.

**Table 2:** Correlation between associated factors and cognitive function.

CERAD		
	ρ	*P*
25(OH)D3	0.091	0.028
Age	− 0.027	< 0.001
Male sex	0.087	0.036
BMI	0.123	0.003
Hemoglobin	0.031	0.456
ALB	0.039	0.346
BUN	0.09	0.032
Creatinine	− 0.062	0.141
eGFR	0.042	0.311
UACR	− 0.019	0.651
Calcium	0.084	0.044
Phosphate	0.097	0.021
Cholesterol	0.044	0.291
Triglyceride	0.021	0.622
Uric acid	0.026	0.531

*CERAD* Consortium to Establish a Registry for Alzheimer’s Disease Word Learning test, *BMI* Body mass index, *ALB* Albumin, *BUN* Blood urea nitrogen, *eGFR* Estimated glomerular filtration rate, *UACR* Urea albumin-creatinine ratio.

### Association between the level of 25(OH) D3 and cognitive function

After propensity score matching, 133 patients were matched in each group. As shown in Table 3, no significant differences were observed between the two groups in age, sex, education level, hemoglobin, serum albumin, calcium, phosphate, creatinine, eGFR, and UACR levels. Multiple logistic regression analysis was used to investigate the relationship between cognitive test and 25(OH) D3 concentration. Table 4 showed that a lower level of 25(OH) D3 was independently associated with a risk of cognitive impairment. The interaction of sex and albuminuria might modify the association between 25(OH)D3 and cognition function (p for interaction = 0.005 and 0.008, respectively), and subgroup analysis was performed in Table 4. In the 30 mg/g ≤ UACR < 300 mg/g level, we found serum 25(OH)D3 was negatively associated with the risk for cognitive impairment. However, a higher level of 25(OH)D3 might not correlate to a better cognitive performance in participants with UACR ≥ 300 mg/g. Our results inferred that a higher level of 25(OH) D3 specifically benefited the cognitive function of patients with microalbuminuria. In addition, an elevated 25(OH) D3 level was associated with a better cognition performance in old male patients with CKD, but not in female patients.

**Table 3:** Comparison of patient characteristics between patients with and without cognitive impairment after propensity score matching.

	No cognitive impairment (n = 133)	Cognitive impairment (n = 133)	*P*
Age (years)	74 (68–80)	74.5 (65.25–80)	0.665
Male (%)	50.4	56.7	0.276
Race (%)			0.451
Mexican American (%)	13.5	11.6	
Other Hispanic (%)	7.5	9.8	
Non-Hispanic White (%)	43.6	42.1	
Non-Hispanic Black (%)	27.8	23.2	
Other (%)	7.5	13.4	
Education (%)			0.531
Less than 9th grade (%)	21.8	26.8	
9 − 11th grade (%)	20.3	17.7	
High school graduate (%)	24.8	26.8	
College or AA degree (%)	20.3	14.6	
College graduate or above (%)	12.8	12.8	
Drink (%)	51.9	55.6	0.54
Hypertension (%)	74.4	73.8	0.898
Diabetes (%)	51.1	46.3	0.412
BMI (kg/m2)	29.34 (25.79–33.97)	27.75 (24.19–32.08)	0.072
Hemoglobin (g/dl)	13.4 (12.5–14.25)	13.5 (12.3–14.5)	0.524
Albumin (g/l)	40 (39–43)	41 (39–43)	0.274
Creatinine (μmol/l)	95.47 (73.37–119.34)	91.94 (73.37–119.34)	0.728
Calcium (mmol/l)	2.35 (2.3–2.43)	2.33 (2.3–2.43)	0.167
Phosphate (mmol/l)	1.2 (1.1–1.32)	1.16 (1.07–1.29)	0.172
eGFR (ml/min/1.73m2)	62.91 (48.63–75.75)	66.56 (47.86–84.09)	0.364
UACR (mg/g)	89.8 (47.83–321.93)	82.86 (42.82–225.65)	0.311
CERAD score	26 (22–30)	14 (9–17)	< 0.001
25(OH)D3 (nmol/l)	64.8 (44.55–93.65)	54.18 (39.76–72.98)	0.003

Data are presented as medians (interquartile ranges) for continuous variables.

*CERAD* Consortium to Establish a Registry for Alzheimer’s Disease Word Learning test, *BMI* Body mass index, *eGFR* Estimated glomerular filtration rate, *UACR* Urea albumin-creatinine ratio; BUN, blood urea nitrogen.

**Table 4:** Logistic regression analysis for cognitive test and 25(OH)D3.

Total	Unadjusted		Adjusted	
	CERAD		CERAD	
	OR (95% CI)	*P*	OR (95% CI)	*P*
Quartile of 25(OH) D3				
Q1	Reference		Reference	
Q2	1.28 (0.679–2.416)	0.445	1.428 (0.692–2.95)	0.335
Q3	0.783 (0.404–1.517)	0.468	0.803 (0.378–1.703)	0.567
Q4	0.411 (0.209–0.807)	0.01	0.417 (0.187–0.928)	0.032
30 mg/g ≤ UACR < 300 mg/g (n = 203)				
Quartile of 25(OH) D3				
Q1	Reference		Reference	
Q2	1.152 (0.536–2.472)	0.717	1.151 (0.49–2.704)	0.746
Q3	1.145 (0.529–2.482)	0.731	1.236 (0.532–2.873)	0.623
Q4	0.344 (0.159–0.744)	0.007	0.385 (0.158–0.938)	0.036
UACR ≥ 300 mg/g (n = 63)				
Quartile of 25(OH) D3				
Q1	Reference		Reference	
Q2	0.857 (0.226–3.249)	0.821	1.035 (0.165–6.478)	0.971
Q3	0.424 (0.115–1.562)	0.197	0.292 (0.041–2.054)	0.216
Q4	0.75 (0.203–2.77)	0.666	0.559 (0.093–3.351)	0.524
Male (n = 140)				
Quartile of 25(OH) D3				
Q1	Reference		Reference	
Q2	0.592 (0.227–1.543)	0.283	0.633 (0.21–1.903)	0.415
Q3	0.65 (0.24–1.759)	0.396	0.538 (0.166–1.741)	0.301
Q4	0.203 (0.073–0.564)	0.002	0.225 (0.067–0.754)	0.016
Female (n = 126)				
Quartile of 25(OH) D3				
Q1	Reference		Reference	
Q2	1.799 (0.653–4.958)	0.256	2.595 (0.711–9.469)	0.149
Q3	1.012 (0.404–2.532)	0.98	1.387 (0.439–4.383)	0.577
Q4	0.726 (0.297–1.733)	0.482	0.628 (0.196–2.014)	0.434

Adjust for age, sex, education, smoke, drink, hypertension, diabetes, BMI, hemoglobin, albumin, BUN, calcium, phosphate, UACR, and eGFR.

*OR* Odds ratio, *CI* Confidence interval, *CERAD* Consortium to Establish a Registry for Alzheimer’s disease.

## Discussion

In this study, we investigated the association between serum 25(OH)D3 and cognitive function in CKD patients with albuminuria. The main finding of this study is that serum levels of 25(OH)D3 positively correlated with cognitive function in patients with CKD, especially in patients with microalbuminuria (30 mg/g ≤ UACR < 300 mg/g).

CKD is classified on the basic of category of eGFR and albuminuria^20^. Cognitive impairment is a deficit in one or more key brain functions, such as attention, memory, visuospatial ability, language skills, and execution skills^21^. The prevalence of cognitive impairment is high among patients with albuminuria, when screened with both California Verbal Learning Test and Symbol Digit Modalities Test^22^. A decline of cognitive function is associated with a prolonged hospitalization, and high mortality^23,24^. Accordingly, itis critical to prevent progression of cognitive impairment.

The mechanism for cognitive dysfunction in patients with kidney damage is complex. It is well known that an older age is a risk factor for cognitive frailty and dysfunction^25^. The severity of renal dysfunction is independently correlated with cognitive impairment^26^, probably owing to an accumulation of uric toxins. Vascular injury and various uremic toxins are suspected to induce cognitive impairment in CKD, including uric acid, indoxyl sulfate, and p-cresyl sulfate^27,28^. Several other risk factors have also been linked to cognitive impairment, such as hyperhomocysteinemia, hypercoagulable states, neuroinflammation, oxidative stress, and impaired cerebral blood flow autoregulation^29,30^.

Vitamin D is a fat-soluble steroid that exerts its effects by binding to the vitamin D receptor. Activation of vitamin D takes place in the liver and kidney by enzymatic hydroxylation with generation of 1.25-dihydroxycholecalciferol or 1.25-dihydroxyvitamin D3^31^. In addition to regulating bone metabolism, vitamin D exerts multiple biological actions, such as obesity, cardiovascular, and neurodegenerative diseases^32,33^. A meta-analysis showed an inverse association of 25(OH)D and cardiovascular and all-cause mortality^34^. However, no associations were found between serum 25(OH)D and risk of dementia^35^ or Alzheimer’s disease^36^ . A study by Parveen et al.^37^ even reported an inverse association of 25(OH)D and cognitive function in patients with type 2 diabetes. 25(OH)D3 is regarded as a marker for vitamin D reserve, and its deficiency is prevalent in patients with CKD^38^. However, the exact association between serum 25(OH)D3 and cognitive function in CKD patients remains inconclusive.

In our study, we divided our CKD patients with UACR ≥ 30 mg/g into 2 groups according to the cutoff value of CERAD. The 25(OH)D3 levels were significantly different between patients with and without cognitive impairment. In addition,, 25(OH)D3 was positively associated with cognitive score in patients with CKD. A propensity-score matching approach was applied to further reduce the bias. In the matched group, a lower level of 25(OH)D3 appeared to be an independent associated factor for cognitive dysfunction in patients with albuminuria. Vitamin D receptors are found in the central nervous system involving in a neuroprotective effect^39^. There is robust evidence that vitamin D3 and 1,25(OH)_2_D3 could regulate the toxicity of reactive oxygen species^40,41^, and neurotrophic factors such as nerve growth factor^42^. The hydroxylated vitamin D3 could induce cell differentiation of embryonic hippocampal cells^43^. In Alzheimer’s disease, 1,25(OH)_2_D3 strongly stimulated amyloid-beta clearance while ameliorating inflammation and apoptosis^44,45^. Meanwhile, the association between 25(OH)D3 and neuroprotection was also explored via improving neuroinflammation and endothelial integrity of the blood–brain barrier^46^. Additionally, evidence demonstrated a protective role of active metabolite of vitamin D3 on renin-angiotensin system and vascular endothelial function^47^. Recent studies showed that a low 25(OH)D was associated with reduced volumes of hippocampal subfields, connection deficits^48^ and a large lateral ventricle volume^49^ in elderly people. However, mores studies are needed to uncover the definite relationship between 25(OH)D3 and hippocampal structure to validate our results. .

By subgroup analysis, we found the association of 25(OH)D3 and cognitive impairment was significant in patients with 30 mg/g ≤ UACR < 300 mg/g, but not in patients with UACR ≥ 300 mg/g. Patients with macroalbuminuria are generally older and have more comorbidities in comparison to those with normal urinalysis^50^. Besides, macroalbuminuria represents a poor residual renal function. Patients with macroalbuminuria displayed higher levels of biomarkers for kidney injury, including neutrophil gelatinase-associated lipocalin^51^. Progression of renal dysfunction is accompanied by the steady accumulation of various uremic toxins. Uremia can cause progressive loss of tissue 25(OH)D3 receptor, probably leading to a tissue vitamin D resistance^52^. Namely, an early and comprehensive evaluation of 25(OH)D3 levels should be emphasized in patients with preserved residual renal function to improve cognition function. Interestingly, our research showed that 25(OH)D3 correlated to a better cognition performance only in male patients, but not in female patients. A supplementing with 25(OH)D3 might increase serum 25(OH)D3 and testosterone level in obese mice^53^. Bioavailable testosterone presented a positive association of cognitive function in older men^54^, while prolactin, progesterone and estradiol might have deleterious effects on cognitive function in older females after menopause^55,56^. However, the number of patients in our subgroup is relatively limited, and the insignificance may be due to the small sample size. Other larger longitudinal studies are needed to confirm our results.

Our study explored a beneficial association of higher levels of 25(OH)D3 and cognitive impairment in CKD patients. Although the enrolled patients were matched with the propensity scores to reduce potential selection bias, there were still certain limitations in the study. This study was a cross-sectional study design which cannot identify causal causation between two factors. In addition, only CERAD test was included in this study, and our results might lead to a bias for other domains of cognitive function. Although this study has adjusted for multiple covariates and a propensity matching was used, more other potential unmeasured confounders should be included in future studies with larger samples.

## Conclusion

Our results suggest that a higher level of serum 25(OH)D3 correlate to a better cognitive performance in CKD patients. With the progression of renal function impairment, a routinely screen and correcting 25(OH)D3 deficiency should be emphasized in the early-stage of CKD. More prospective studies are necessary to confirm the protective effects of 25(OH)D3 supplements on cognition in CKD patients.

## Methods

### Study population

For the present study, we analyzed secondary data from the 2011 to 2012 and 2013 to 2014 NHANES. The National Health and Nutrition Examination Survey (NHANES) is a large, complex, survey of US population conducted by National Center for Health Statistics to provide nationally representative estimates on the health and nutritional status. The NHANES protocol was approved by the National Center for Health Statistics (NCHS) Research Ethics Review Board (http://www.cdc.gov/nchs/nhanes). The study was conducted in accordance with the relevant guidelines and regulations of the Declaration of Helsinki. All the NHANES participants provided with informed consent.

**Figure 1:**
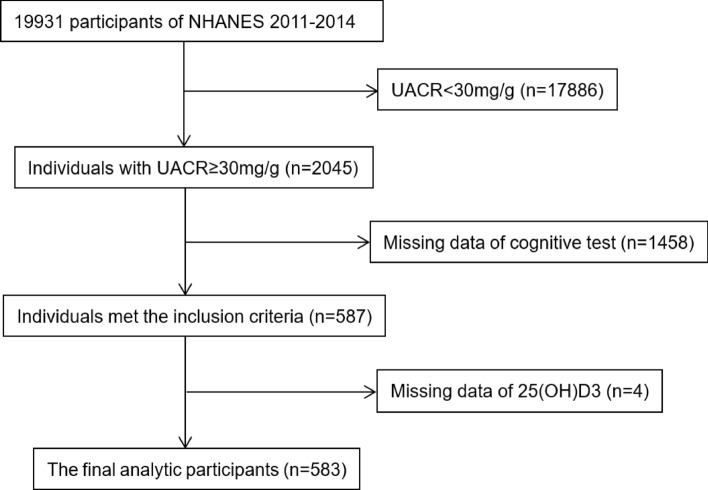
A flow chart of screening.

A total of 2045 participants with albuminuria (UACR ≥ 30 mg/g) were included. Serum creatinine was used to calculate eGFR with the Modification of Diet in Renal Disease (MDRD) equation. The CKD stages were defined with eGFR and/or evidence of kidney damage according to the KDIGO guidelines: stage 1, eGFR ≥ 90 mL/min/1.73 m^2^ with UACR ≥ 30 mg/g; stage 2, eGFR 60–89 mL/min/1.73 m2 with UACR ≥ 30 mg/g; stage 3, eGFR 30–59 mL/min/1.73 m2; stage 4, eGFR 15–29 mL/min/1.73 m2; stage 5, eGFR < 15 mL/min/1.73 m^2^^57^. Among them, 587 individuals ≥ 60 years old underwent cognitive assessment. Participants were further excluded due to missing data for 25(OH)D3 levels. Finally, 583 participants were recruited for the subsequent analysis (shown in Fig. 1).

### Measurement of serum 25(OH)D3

In the present study, we extracted serum 25(OH)D3 from the database. In the 2011–2014 cycle of the NHANES, serum 25(OH)D3 concentrations were measured by a standardized ultra-high performance liquid chromatography–tandem mass spectrometry method to quantitatively detect 25(OH)D3.

### Cognitive function assessment

The Consortium to Establish a Registry for Alzheimer’s Disease Word Learning subtest (CERAD) was used to assess participants’ cognitive function. CERAD assesses immediate and delayed learning ability for new verbal information (memory sub-domain)^58^. The test consists of three consecutive learning trials and a delayed recall trial. For each trial, participants were asked to read ten random words and recall as many words as they could immediately. According to the previous papers, we used the 25th percentile of the scores as the cutoff points^59^. Thus, participants were divided into low and normal cognition performance groups.

### Covariates

The sociodemographic variables and health condition information were obtained, including age, sex, education level, smoking (yes/no), alcohol consumption (yes/no), hypertension (yes/no), and diabetes (yes/no). Body mass index (BMI) is calculated by dividing weight in kilograms by the square of height in meters. Serum albumin, calcium, phosphate, alkaline phosphatase, creatinine, blood urea nitrogen (BUN), cholesterol, triglyceride, and uric acid were also collected.

### Statistical analysis

All the data were analyzed by SPSS 23.0 statistical software. Baseline characteristics of patients were reported using percentages for categorical variables, and medians (interquartile range, IQR) for continuous data. Continuous variables and categorical variables were compared among groups using Kruskal–Wallis and χ2 test, separately. The relationship between cognitive score and serum 25(OH)D3 level were assessed using the Spearman’s correlation analysis for non-normally distributed. To identify a more accurate relationship between cognition and 25(OH)D3, propensity matching analysis was performed. Patients in the cognition impairment group were matched at a ratio of 1:1 with those in the non-cognition impairment group by propensity scores. Variables age, sex, education level, race, smoke, drink, hypertension, diabetes, hemoglobin, albumin, BUN, eGFR, UACR, calcium, and phosphate were selected for the propensity matching model. Logistic regressions were performed to assess the association between cognitive function (dependent variable) and serum 25(OH)D3 (independent variable). Confounding variables were selected based on clinical relevance and a *p* < 0.05 in the correlation analysis. Subgroup analysis was performed according to the UACR levels and sex. In detail, populations were grouped into 30 mg/g ≤ UACR < 300 mg/g (microalbuminuria) and UACR ≥ 300 mg/g (macroalbuminuria) groups^60^. Statistical hypotheses were tested using a two-tailed *P* ≤ 0.05 level of significance.

### Ethics approval

All participants submitted written informed consent and the study was approved by the NCHS Research Ethics Review Board (Continuation of Protocol #2011–17). The studies were conducted according to the guidelines of the Declaration of Helsinki throughout.

### Supplementary Information

Supplementary Table 1.

## Data Availability

The datasets generated and analyzed in the present study are available on the website of NHANES datasets 2011–2014 (https://wwwn.cdc.gov/nchs/nhanes).
