# Supplementary material for: Association between serum 25-hydroxyvitamin D3 level and cognitive impairment in older chronic kidney disease patients
Source: Sci Rep. 2024 May 30;14:12403. doi: 10.1038/s41598-024-63350-y (PMC11137016; doi:10.1038/s41598-024-63350-y)
Supplement: Supplementary file 1 — Supplementary Table 1. [file 41598_2024_63350_MOESM1_ESM.pdf]

Supplementary Table 1 Clinical characteristics of subjects with urinary albuminuria in National Health and Nutrition Examination Survey (NHANES) 2011−2014 (Stratified by 25(OH)D3 Quartiles)

	Total (n=584)	Quartile 1 (n=146)	Quartile 2 (n=146)	Quartile 3 (n=146)	Quartile 4 (n=146)	P
Age	71 (65-80)	67 (62-76)	70 (65-78)	72 (66-80)	75 (68-80)	<0.001
Male (%)	49.4	48.6	61	52.7	46.6	0.069
Race (%)						<0.001
Mexican American (%)	10.5	14.4	15.1	4.8	8.2	
Other Hispanic (%)	9.9	8.9	9.6	12.3	7.5	
Non-Hispanic White (%)	43.8	29.5	37.7	51.4	58.9	
Non-Hispanic Black (%)	26.1	39.7	26	21.2	17.1	
Other (%)	9.8	7.5	37.6	10.3	8.2	
Smoke (%)	33.4	45.2	28.8	35.6	31.5	0.012
Drink (%)	57.5	63	59.6	57.5	63	0.435
Hypertension (%)	75.4	75.3	75.3	73.3	73.3	<0.001
Diabetes (%)	41.8	50	41.1	39.7	39.7	<0.001
Education (%)						0.049
Less than 9th grade (%)	15.2	17.1	19.2	13.7	14.4	
9−11th grade (%)	16	19.2	19.2	15.8	13.7	
High school graduate (%)	23.2	26	28.1	23.3	20.5	
College or AA degree (%)	23.8	28.1	16.4	22.6	33.6	
College graduate or above (%)	15.7	8.9	16.4	24	17.1	
CKD stage (%)						0.02
CKD stage 1	25.1	21.2	19.9	28.8	13.7	
CKD stage 2	39.5	45.9	41.8	39.7	39.7	
CKD stage 3	27.9	21.2	32.2	26.7	37.7	
CKD stage 4	5.2	7.5	3.4	3.4	7.5	
CKD stage 5	2.3	4.1	2.7	1.4	1.4	
Hemoglobin (g/dl)	13.5 (12.5-14.5)	13.5±1.62	13.69±1.67	13.53±1.65	13.34±1.37	0.108
Albumin (g/l)	42 (39-43)	40.5 (38-43)	41 (39-43)	42 (40-44)	42 (40-44)	0.002
BUN (mg/dl)	11 (6.07-17)	11 (5.36-16)	10.18 (5.71-18)	10 (5.71-16)	12 (7.5-18.46)	0.047
Creatinine (μmol/l)	90.17 (71.6-117.79)	86.19 (71.6-112.93)	94.59 (72.93-115.58)	83.1 (68.95-110.06)	99.01 (74.26-123.32)	0.052

Calcium (mmol/l)	2.35 (2.3-2.43)	2.35 (2.3-2.43)	2.34 (2.28-2.4)	2.35 (2.3-2.41)	2.38 (2.33-2.43)	0.003
Phosphate (mmol/l)	1.2 (1.1-1.32)	1.2 (1.1-1.36)	1.2 (1.01-1.32)	1.2 (1.01-1.31)	1.2 (1.1-1.36)	0.525
Alkaline phosphatase (u/l)	72 (56-89)	74 (61.25-92)	73.5 (55-93.75)	71 (56-88)	66 (54-82)	0.02
Cholesterol (mmol/l)	4.66 (3.91-5.61)	4.62 (3.91-5.77)	4.58 (3.76-5.68)	4.66 (3.87-5.59)	4.76 (4.09-5.43)	0.625
Triglyceride (mmol/l)	1.54 (1.04-2.43)	1.56 (1.01-2.47)	1.52 (1.02-2.44)	1.65 (1.17-2.42)	1.49 (0.97-2.38)	0.476
Uric acid (mg/dl)	5.9 (4.9-7.2)	5.75 (4.7-7.2)	5.85 (4.7-7.18)	5.9 (5-6.7)	6.25±1.67	0.447
eGFR (ml/min/1.73m2)	67.05 (51.63-85.1)	68.42±27.69	69.42±28.53	72.49±26.07	62.71±23.14	0.058
UACR (mg/g)	69.68 (43.68-170.32)	97.79 (43.57-280.07)	67 (41.82-142.15)	56.19 (41.21-123.75)	81.39 (47.74-161.09)	0.099
CERAD score	23 (17-28)	24 (17-27)	22 (16-27.25)	23 (18-28)	24.5 (20.75-28.25)	0.023
25(OH)D3 (nmol/l)	61.56 (41.84-84.98)	29.46 (21.75-36.05)	52 (46.68-57.51)	72.8 (66.07-79.13)	99.42 (91.15-121.26)	<0.001

AF, Animal Fluency test; CERAD-WL, Consortium to Establish a Registry for Alzheimer's Disease Word Learning test; CERAD-DR, Consortium to Establish a Registry for Alzheimer's Disease Delayed Recall test; DSST, Digit Symbol Substitution test; CKD, chronic kidney disease; eGFR, estimated glomerular filtration rate; UACR, urea albumin-creatinine ratio; BUN, blood urea nitrogen.
